# The Fitting and Furnishing of Wards and Operating Theatres

**Published:** 1906-11-03

**Authors:** 


### THE FITTING AND FURNISHING OF WARDS AND OPERATING THEATRES.

### DISPENSARIES AND OUT-PATIENT DEPARTMENTS.

The principal difficulty in the out-patient department of a large hospital is to efficiently ventilate, and to guard against the formation of beds of dirt and the harbouring of germs. In many of the leading hospitals the plenum system of ventilation has been established for the out-patient department, plant being laid down specially for the purpose, though it is not taken into the wards. In the latest out-patient departments the arrangement of the ventilation of the halls and the surgeons' consulting-rooms is by inlets, either consisting of gratings fixed about eight feet above the floor, or of open-mouthed ducts, forming part of ornamental pillars, the mouths of the ducts being arranged so that the air on entering the hall is directed upwards. The exhaust air from the consulting-rooms is sometimes led into the hall, the whole of the vitiated air from both being led away together. Where the entry ducts are placed above the heads of those in the hall, or where it is directed over their heads, the exit ducts are placed near the floor, immediately under the entry grating, or duct, this being the view of modern heating and ventilating engineers, is the best course for the air. There is considerable controversy between different schools of heating and ventilating engineers, as usual, as to whether it is best for the vitiated air to be taken out near the ceiling, or near the floor. All those who have worked at the plenum system, however, appear to be agreed that the latter is best. In the out-patient hall of the Birmingham General Hospital the entry and exit ducts are arranged in the pillars of the hall to look like ornamental gratings. The effect in the hall itself is very good, but the surgeons complained that at times the air got stuffy in their consulting-rooms. This, however, was explained by the superintendent to be caused by the fact that, as in so many cases, the number treated far exceeded that originally intended, hence the apparatus proved in such cases too small for the work.

In the Birmingham General Hospital it was found necessary to place boards in certain positions at the ends in the out-patient hall, to guide the air into the hall, so as to obtain the full benefit of the whole of the ventilating current. It was also found necessary to place guiding boards in the basement to direct the air current into a ward, where a duct leading to the ward opened out of a large duct, carrying a quantity of air past the entrance to the ward duct. The same "injector" action was present that was referred to in connection with the ventilation of the sanitary blocks of the wards, the friction of the air current across the mouth of the ward duct caused a motion of air from the ward instead of into it. A small guiding-board easily put the matter right. Floors of out-patient departments in the leading hospitals are usually of terrazzo, or one of the substances that has been introduced since aseptic treatment came into vogue. There are several of these, made from various materials, the virtues of all of them being that they present a smooth surface, which can easily be cleaned and does not harbour germs. Terrazzo has the trick of cracking, but the makers of the substance claim that the cracks can easily be made good. In many instances the floors are laid in panels, so that if a crack takes place, a panel can be relaid. Walls of out-patient departments are either of glazed tiles—some very beautiful designs by Messrs. Doulton and others having been used for this purpose—of glass tiles, or of cement, overlaid with one of the enamel paints on the market. The junction of the walls with the floor is always made with a curve, and not with a corner, for the collection of dust, and it is claimed as an advantage, for some of the substances that have been put on the market, such as Segalith, Eubeolith and others, to take the place of terrazzo, and which can be used for walls as well as floors, that there can be a complete absence of junctions, the whole mass being laid at one operation.

### Dispensaries.

The tendency in a few leading hospitals is to make more and more of the materials dispensed in the hospital itself. It is not so much the cost, though in a large hospital that may be a consideration, but it is claimed that by purchasing the herbs and other substances at first hand, and submitting them to careful analysis, both when they arrive and from time to time while they are in stock, the physician has a better chance that his prescriptions have fair play. On the other hand, the great drug companies claim that they have such a large sale and quick distribution that their drugs are always fresh. It does not pay to lay down a machine unless the economy it brings is sufficient to pay all charges incident to its establishment, within the term of its useful life, and machines are constantly being improved. At the London Hospital nearly the whole work of preparing the medicines is done in the dispensary; pill machines, rounders, coaters, tabloid machines, lozenge machines, sifters for powder-mixers, tincture presses, etc., are run by means of electric motors taking current from the Stepney Borough supply service; as well as a disintegrator for grinding up roots. The ordinary chemical work of a drug laboratory is carried on in the dispensaries of most large hospitals; steam, which is nearly always available, forming a handy method of heating, steam jacketted boiling, and evaporating pans being employed. Several hospitals also make their own soda-water, and here, at any rate, there appears to be no doubt as to the economy. Liquefied carbonic acid is allowed to enter a mixing-cylinder, into which a spray of water is also forced, the water in its finely-divided state taking up a large percentage of the carbonic acid with the aid of revolving paddles in the mixing cylinder. This is something similar to the method employed by Schweppes. At the London Hospital a soda-water machine made by Messrs. Hayward Tyler and Co., and driven by a 5 h.p. electric motor, makes 48 dozen siphons per day, throughout the year, 70 dozen a day being made in the summer months, at a cost of 1¼d. per dozen siphons, all told. Cold storage might very well be employed in the dispensary by means of cold brine carried in pipes, from the refrigerating apparatus, where there is a cold storage plant on the ground.

The large out-patient hall at the London Hospital is ventilated by fans in the roof exhausting the air, the inlets being by tobin tubes from outside.

